# Complexes of 2,4,6-tri­hydroxy­benzoic acid: effects of intra­molecular hydro­gen bonding on ligand geometry and metal binding modes

**DOI:** 10.1107/S2053229622009901

**Published:** 2022-10-25

**Authors:** Brendan F. Abrahams, Christopher J. Commons, Timothy A. Hudson, Robin Sanchez Arlt, Rion Ahl, Eirene D. Carajias, Jason W. K. Chan, Zhihao Guo, Renee E. Hill, Alice McGinty, Neale L. Peters, Joshua Y. P. Poon, Jingqi Qu, Jinglin Qu, Emily E. Rochette, Catherine Walkear, Hanlin Wang, Holly Wu, Chang Xu, Jingyuan Zhang

**Affiliations:** a School of Chemistry, University of Melbourne, Parkville, VIC 3010, Australia; b Scotch College, 1 Morrison Street, Hawthorn, VIC 3122, Australia; c Melbourne Girls’ College, Yarra Boulevard, Richmond, VIC 3121, Australia; d Melbourne Graduate School of Education, University of Melbourne, Parkville, VIC 3010, Australia; Australian National University, Australia

**Keywords:** car­box­yl­ate, crystal engineering, crystallographic education, crystal structure, 2,4,6-tri­hydroxy­benzoic acid, intra­molecular hydro­gen bonds, coordination polymers

## Abstract

This article describes a series of more than 20 new compounds formed by the combination of 2,4,6-tri­hydroxy­benzoic acid (H_4_thba) with metal ions in the presence of a base, with structures that include discrete mol­ecular units, chains, and two- and three-dimensional networks. As a result of the presence of two *ortho*-hy­droxy groups, H_4_thba is a relatively strong acid (p*K*_a1_ = 1.68). The car­box­yl­ate group in H_3_thba^−^ is therefore considerably less basic than most car­box­yl­ates with intra­molecular hydro­gen bonds, conferring a rigid planar geometry upon the anion. These characteristics of H_3_thba^−^ significantly impact upon the way it inter­acts with metal ions. In *s*-block metal compounds, where the inter­action of the metal centres with the car­box­yl­ate O atoms is essentially ionic, the anion bonds to up to three metal centres *via* a variety of binding modes. In cases where the metal ion is able to form directional coordinate bonds, however, the car­box­yl­ate group tends to bond in a mono­den­tate mode, inter­acting with just one metal centre in the *syn* mode. A dominant influence on the structures of the complexes seems to be the face-to-face stacking of the aromatic rings, which creates networks containing layers of metal–oxygen polyhedra that participate in hydro­gen bonding. This investigation was undertaken, in part, by a group of secondary school students as an educational exercise designed to introduce school students to the technique of single-crystal X-ray diffraction and enhance their understanding of primary and secondary bonding.

## Abstract 2

More than 20 new compounds derived from 2,4,6-tri­hydroxy­benzoic acid (H_4_thba) have been synthesized, with structures that include discrete mol­ecular units and chains, in addition to two- and three-dimensional nets. Intra­molecular hydro­gen bonds between the *ortho*-hy­droxy groups and the car­box­yl­ate group in the H_3_thba^−^ anion confer a rigid geometry upon the ligand which, when combined with the low basicity of the car­box­yl­ate group, limits the variety of metal-binding modes.

## Introduction

**Figure 1:**
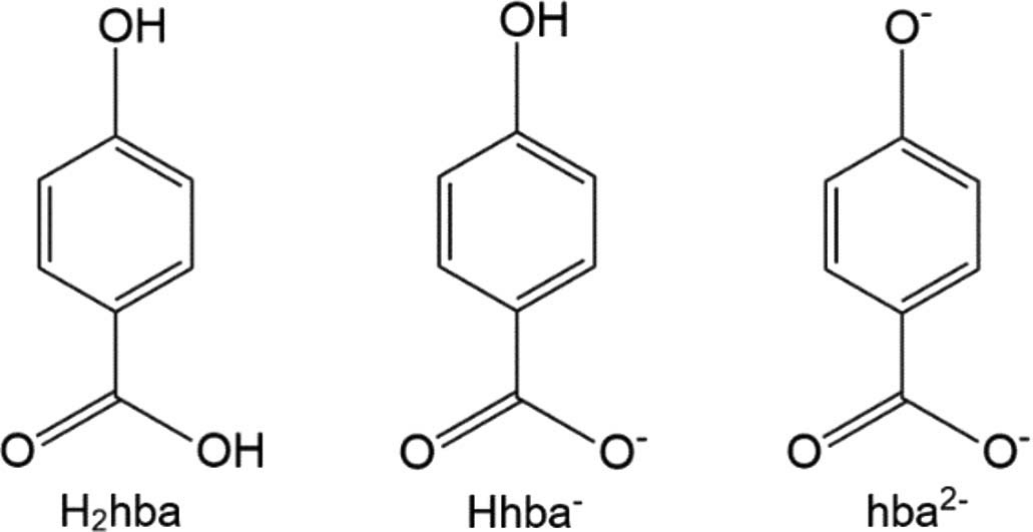
4-Hy­droxy­benzoic acid (H_2_hba) and its monoanion Hhba^−^ and dianion hba^2−^.

A recent article (Abrahams *et al.*, 2021 ▸) describes the structures of alkali metal salts of 4-hy­droxy­benzoic acid (H_2_hba). Whilst H_2_hba is a relatively simple organic mol­ecule, it displays remarkable variation in its binding to metal centres. It reacts with Group 1 metal hydroxides in aqueous solution to form ionic solids containing either the uncharged mol­ecule, the monoanion Hhba^−^ (4-hy­droxy­ben­zo­ate) or the dianion hba^2−^ (4-oxidoben­zo­ate) (Fig. 1 ▸).

**Figure 2:**
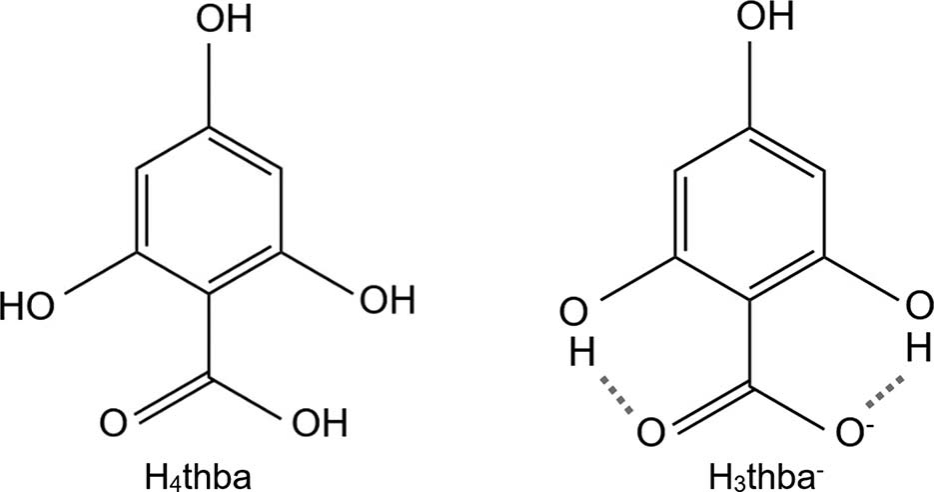
2,4,6-Tri­hydroxy­benzoic acid (H_4_thba) and its monoanion H_3_thba^−^. In the anion, hydro­gen bonds are present between the O atoms of the car­box­yl­ate group and the H atoms of the *ortho*-hy­droxy groups.

This article describes the results of a study of the complexes of 2,4,6-tri­hydroxy­benzoic acid [H_4_thba; Fig. 2 ▸(*a*)] and its anionic forms. The presence of additional hy­droxy groups in H_4_thba compared with H_2_hba offers the prospect of a greater diversity of coordination modes to metal ions, together with the potential for formation of hydro­gen bonds that could aid in the generation of inter­esting supra­molecular structures.

It is rather surprising that no metal complexes of H_4_thba or its anions are included in the Cambridge Structural Database (CSD, Version 5.43, June 2022 release; Groom *et al.*, 2016 ▸), given the inter­est in the use of aromatic car­box­yl­ates as linkers in the synthesis of coordination polymers for technologies such as gas storage, catalysis and separation. Structures have been reported for the cocrystals of H_4_thba with water, pyrazine and bis­phenazine (Jankowski *et al.*, 2007 ▸), and for salts with organic ammonium cations (Dandela *et al.*, 2018 ▸; Ganduri *et al.*, 2019 ▸; Jankowski *et al.*, 2007 ▸; Mittapalli *et al.*, 2015 ▸; Prior *et al.*, 2009 ▸; Sarmah *et al.*, 2020 ▸; Seaton, 2014 ▸).

H_4_thba is a remarkably strong carb­oxy­lic acid (p*K*_a1_ = 1.68; Dean, 1999 ▸), with a similar strength to sulfurous acid. It is much more acidic than H_2_hba (p*K*_a1_ = 4.5) and benzoic acid (p*K*_a_ = 4.2). The relatively high acidity of H_4_thba arises from strong intra­molecular hydro­gen bonds that form between the two *ortho*-hy­droxy groups and the car­box­yl­ate group in the H_3_thba^−^ ion (Fig. 2 ▸), which stabilize the conjugate car­box­yl­ate base.

**Figure 3:**
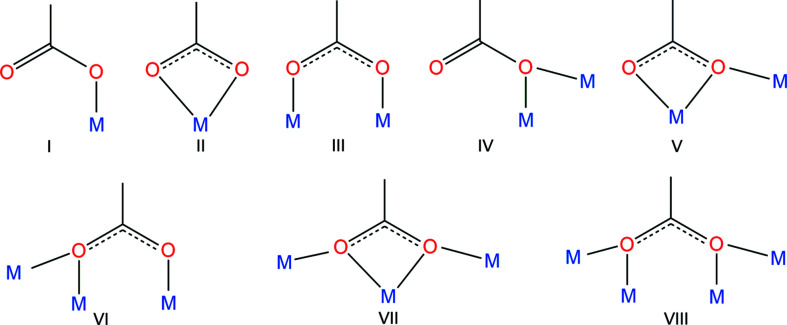
Examples of coordination modes of car­box­yl­ate ligands.

Carboxyl­ates exhibit a wide variety of coordination modes. Whilst the car­box­yl­ate anion can bind as a chelating ligand, the strain associated with the formation of the four-membered chelate ring often results in the adoption of different coordination modes, many of which involve inter­actions with multiple metal centres. Some of the more common coordination modes are depicted in Fig. 3 ▸ (Hu *et al.*, 2011 ▸; Rardin *et al.*, 1991 ▸). In the case of the complexes formed from H_2_hba, for example, modes I, III, IV, VI and VIII have been observed (Abrahams *et al.*, 2021 ▸, 2022 ▸; White *et al.*, 2015 ▸). In view of the relatively high acidity of H_4_thba, the car­box­yl­ate group in the H_3_thba^−^ ion is much less basic than in, for example, the ben­zo­ate ion and Hhba^−^.

The coordination of a car­box­yl­ate group to an individual metal centre can be classified as being either *syn* or *anti* (Ryde, 1999 ▸). In the *syn* configuration, the second O atom of the car­box­yl­ate group is on the same side of the C—O bond as the metal centre. In this instance, the *M*—O—C—O torsion angle will be close to 0°. In the *anti* configuration, the second O atom of the car­box­yl­ate group is on the opposite side of the C—O bond as the metal centre and the *M*—O—C—O torsion angle will be close to 180°. The most common configuration for car­box­yl­ates is the *syn* form, although numerous examples of the *anti* form exist in the literature. For the complexes formed from H_4_thba, it was anti­cipated that the presence of the *ortho*-hy­droxy groups would restrict coordination to the *syn* con­figuration. Significant deviation from *M*—O—C—O torsion angles of 0 or 180° may be expected when the inter­action between the metal cation and the car­box­yl­ate is purely ionic.

This investigation was performed, in part, as a seven-week elective research program for secondary school students. The 12 students who participated were in the penultimate year of secondary education (Year 11; average age 16 years) and attended Melbourne Girls’ College and Scotch College Melbourne. The program aimed to introduce students to the power of the technique of X-ray crystallography, a topic that is unfortunately missing from many modern introductory secondary school chemistry courses. The students attended weekly one-hour sessions covering the basic principles of crystallography, including the use of the *OLEX2* software package (Dolomanov *et al.*, 2009 ▸), and then performed experimental work to make new crystalline compounds in the school laboratory. In a few instances, when reactions were considered unsuitable for students to perform, the synthetic work was performed by University of Melbourne researchers.

The compounds derived from 2,4,6-tri­hydroxy­benzoic acid, C_7_H_6_O_5_, described here are: di-μ-aqua-bis­[tri­aqua­(2,4,6-tri­hy­droxy­ben­zo­ato)lithium] dihydrate, [Li_2_(C_7_H_5_O_5_)_2_(H_2_O)_8_]·2H_2_O, **1**, poly[μ-aqua-μ-2,4,6-tri­hydroxy­ben­zo­ato-potassium], [K(C_7_H_5_O_5_)(H_2_O)]_*n*_, **2**, poly[hemi­aqua-μ-2,4,6-tri­hydroxy­ben­zo­ato-rubidium], [Rb_2_(C_7_H_5_O_5_)_2_(H_2_O)]_*n*_, **3**, poly[μ-2,4,6-tri­hydroxy­ben­zo­ato-caesium], [Cs(C_7_H_5_O_5_)]_*n*_, **4**, poly[μ-aqua-(μ-2,4,6-tri­hydroxy­ben­zo­ato)(μ-2,4,6-tri­hydroxy­benzoic acid)caesium], [Cs(C_7_H_5_O_5_)(C_7_H_6_O_5_)(H_2_O)]_*n*_, **5**, hexa­aqua­mag­nes­ium(II) bis­(2,4,6-tri­hydroxy­ben­zo­ate) dihydrate, [Mg(H_2_O)_6_](C_7_H_5_O_5_)_2_·2H_2_O, **6**, guanidinium 2,4,6-tri­hydroxy­ben­zo­ate monohydrate, [C(NH_2_)_3_][C_7_H_5_O_5_]·H_2_O, **7**, di-μ-aqua-di-μ-2,4,6-tri­hydroxy­ben­zo­ato-bis­[tetra­aqua­calcium(II)] bis­(2,4,6-tri­hydroxy­ben­zo­ate) tetra­hydrate, [Ca_2_(C_7_H_5_O_5_)_2_(H_2_O)_10_](C_7_H_5_O_5_)_2_·4H_2_O, **8**, poly[tetra­aqua­bis­(μ-2,4,6-tri­hydroxy­ben­zo­ato)strontium], [Sr(C_7_H_5_O_5_)_2_(H_2_O)_4_]_*n*_, **9**, poly[tetra­aquabis­(μ-2,4,6-tri­hydroxy­ben­zo­ato)barium], [Ba(C_7_H_5_O_5_)_2_(H_2_O)_4_]_*n*_, **10**, poly[[tetra­aqua­(μ-2,4,6-tri­hydroxy­ben­zo­ato)bis­(2,4,6-tri­hy­droxy­ben­zo­ato)cerium(III)] dihydrate], {[Ce(C_7_H_5_O_5_)_3_(H_2_O)_4_]·2H_2_O}_*n*_, **11**, tetra­aqua­bis­(2,4,6-tri­hydroxy­ben­zo­ato)man­gan­ese(II) tetra­hydrate, [Mn(C_7_H_5_O_5_)_2_(H_2_O)_4_]·4H_2_O, **12** and **13**, tetra­aqua­bis­(2,4,6-tri­hydroxy­ben­zo­ato)cobalt(II) tetra­hydrate, [Co(C_7_H_5_O_5_)_2_(H_2_O)_4_]·4H_2_O, **14**, tetra­aqua­bis­(2,4,6-tri­hy­droxy­ben­zo­ato)nickel(II) tetra­hydrate, [Ni(C_7_H_5_O_5_)_2_(H_2_O)_4_]·4H_2_O, **15**, tetra­aqua­bis­(2,4,6-tri­hydroxy­ben­zo­ato)zinc(II) tetra­hydrate, [Zn(C_7_H_5_O_5_)_2_(H_2_O)_4_]·4H_2_O, **16**, *catena*-poly[[bis­(2,4,6-tri­hy­droxy­ben­zo­ato)copper(II)]-di-μ-aqua], [Cu(C_7_H_5_O_5_)_2_(H_2_O)_2_]_*n*_, **17**, *catena*-poly[[[bis­(2,4,6-tri­hydroxy­ben­zo­ato)cadmium(II)]-di-μ-aqua] penta­hydrate], {[Cd(C_7_H_5_O_5_)_2_(H_2_O)_2_]·5H_2_O}_*n*_, **18**, hexa­aqua­manganese(II) bis­(2,4,6-tri­hydroxy­ben­zo­ate) dihydrate, [Mn(H_2_O)_6_](C_7_H_5_O_5_)_2_·2H_2_O, **19**, *catena*-poly[aqua­bis­(μ-2,4,6-tri­hydroxy­ben­zo­ato)lead(II)], [Pb(C_7_H_5_O_5_)_2_(H_2_O)]_*n*_, **20**, poly[μ-aqua-tri­aqua-(μ_3_-5-oxo­cyclo­hexa-2,5-diene-1,3-diolato)dilithium], [Li_2_(C_6_H_4_O_3_)(H_2_O)_4_]_*n*_, **21**, and poly[[{μ-(1*S*,2*S*)-1-hy­droxy-2-[(*R*)-1-hy­droxy-2-oxido-4,6-dioxo­cyclo­hex-2-en-1-yl]-3-oxido-5-oxo­cyclo­pent-3-ene-1-car­box­yl­ato}tricaesium] 0.75-hydrate], {[Cs_3_(C_12_H_7_O_9_)(H_2_O)]·0.75H_2_O}_*n*_, **22**.

X-ray crystal data sets were collected at the University of Melbourne and returned to the students by university staff. Under supervision, students determined and refined their crystal structures using *SHELXT* (Sheldrick, 2015*a* ▸) and *SHELXL* (Sheldrick, 2015*b* ▸), respectively, within *OLEX2*.

## Experimental

### Synthesis and crystallization

H_4_thba was combined with the hydroxides of lithium, sodium, potassium, rubidium and caesium in a series of reactions involving different stoichiometric ratios in aqueous solution. Typically, this involved heating 0.10 g (0.58 mmol) of H_4_thba and the appropriate amount of metal hydroxide in 5 ml of warm water (50 °C) until the solids dissolved. Crystals of the alkali metal salts suitable for single-crystal X-ray diffraction formed upon cooling and evaporation of the solvent. Compounds **1**–**3** were formed from 1:1 mixtures of the metal hydroxide and H_4_thba, but they could also be formed from combinations in other proportions.

Compound **4** was prepared from a 1:1 mixture of CsOH and H_4_thba, whilst a 1:2 mixture formed **5**. Compound **21** was prepared from a 4:1 mixture, heated to 50 °C, whilst in the case of compound **22**, the mixture was heated on a hotplate almost to dryness.

The complexes of magnesium, calcium, barium, manganese, copper, cobalt, nickel, zinc, lead and cadmium (**6**, **8**, **10** and **12**–**20**) were obtained by heating 0.10 g (0.58 mmol) of H_4_thba with the corresponding metal acetate in a 1:1 reaction mixture in 5 ml of warm water (50 °C) until the solids dissolved. Crystals formed when the solution cooled and the solvent evaporated. Remarkably, in the case of manganese, three different crystalline products with the same elemental composition were obtained using this procedure (**12**, **13** and **19**).

The strontium salt (**9**) was produced by reacting 0.022 g (0.52 mmol) of LiOH·H_2_O with 0.10 g (0.58 mmol) of H_4_thba in 3 ml of water at room temperature. A 1 ml solution of 0.071 g (0.25 mmol) of Sr(NO_3_)_2_·4H_2_O was added and the mixture was heated at about 50 °C for 5 min. Crystals were obtained by solvent evaporation.

The synthesis of the cerium salt (**11**) followed the same procedure as for the strontium salt, using 0.087 g (0.20 mmol) of Ce(NO_3_)_3_·6H_2_O.

Finally, the guanidinium salt (**7**) was produced by reacting 0.022 g (0.52 mmol) of LiOH·H_2_O with 0.10 g (0.58 mmol) of H_4_thba in 3 ml of water at room temperature. A 7 ml solution of 0.075 g (0.79 mmol) of guanidinium chloride was added and the mixture heated at about 50 °C for 5 min.

Crystals were obtained by solvent evaporation with good yields obtained for each of the reactions. Visual inspection of the crystalline materials indicated a homogenous product in most of the reaction mixtures. Occasionally, different crystal habits were observed; however, these were shown to be the same crystalline material based on unit-cell determinations.

### Refinement

Crystal data, data collection and structure refinement details are summarized in Table 1 ▸. The H atoms of water mol­ecules, hy­droxy groups and carb­oxy­lic acid groups were located in difference Fourier maps and refined using a riding-model approximation, with O—H distances fixed at 0.85 Å and *U*_iso_(H) = 1.5*U*_eq_(O). H atoms bonded to O atoms were not modelled for compound **11**. For the other compounds, non-hy­droxy­lic H atoms were placed in calculated positions and refined as riding atoms, with C—H = 0.95 Å and *U*_iso_(H) = 1.2*U*_eq_(C) for ring H atoms. *R*_int_ values were not given for refinements that involved the use of merged data sets from twinned crystals (*SHELXL* HKLF5 format), *i.e.* those for **3**, **4** and **9**. Details of the refinements can be found in the CIF files.

## Results and discussion

Whilst, in principle, H_4_thba has up to four H atoms that might be lost in the formation of complexes, nearly all the compounds described in this article contain the monoanion, H_3_thba^−^ (2,4,6-tri­hydroxy­ben­zo­ate). The exceptions are com­pounds **21** and **22**, in which the organic components are decomposition products of H_4_thba. The caesium ion was found to associate with both the deprotonated and neutral forms of H_4_thba, yielding compound **5** of composition Cs(H_3_thba)(H_4_thba)(H_2_O).

As described below, H_4_thba reacts to form complexes with a wide range of different and inter­esting structures, including monomers, dimers, chains, and two- and three-dimensional networks. The following descriptions of compounds **1**–**22** will focus on the most significant structural aspects of the crystalline products, with the aim of identifying key factors that determine their structure.

In the descriptions of the structures that follow, the complexes are grouped on the basis of the nature of the bonds to the metal centres (Sections 3.1 and 3.2). This is followed by observations regarding the stability of H_4_thba in its reactions with metal ions (Section 3.3) and finally a discussion of main structural trends (Section 3.4).

### Structure description of complexes with ionic bonds

**Figure 4:**
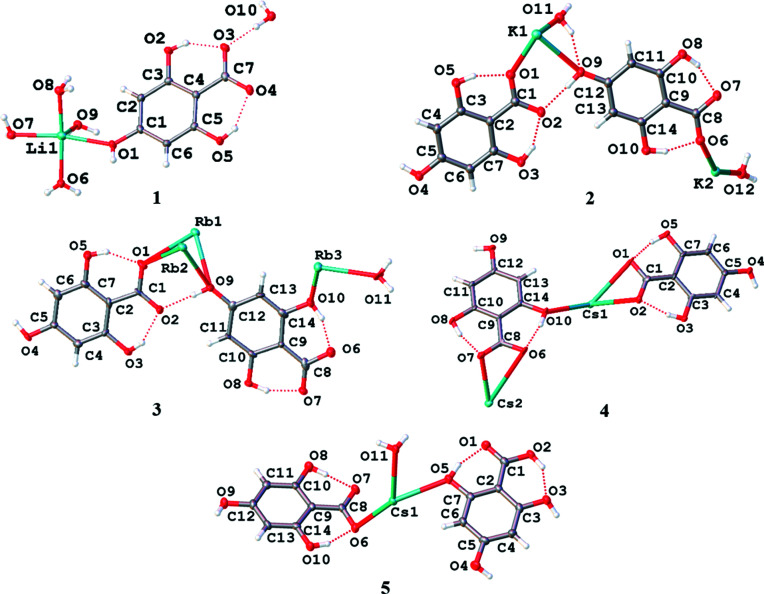
The asymmetric units of Li_2_(H_3_thba)_2_(H_2_O)_8_·2H_2_O, **1**, K(H_3_thba)·H_2_O, **2**, Rb(H_3_thba)·0.5H_2_O, **3**, Cs(H_3_thba), **4**, and Cs(H_3_thba)(H_4_thba)·H_2_O, **5**, showing the atom-labelling schemes for the compounds. In this and later figures of asymmetric units, displacement ellipsoids are represented at the 50% probability level and H atoms are depicted by spheres of arbitrary size. The red dotted lines represent hydro­gen-bonding inter­actions.

The structures of the asymmetric units of the Group 1 metal salts formed from H_4_thba are shown in Fig. 4 ▸.

**Figure 5:**
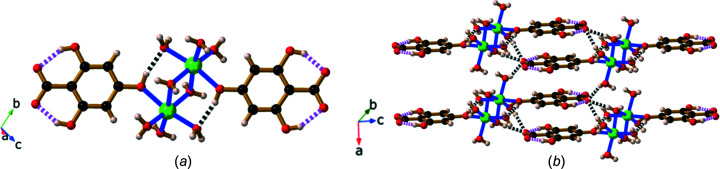
Views of the structure of Li_2_(H_3_thba)_2_(H_2_O)_8_·2H_2_O (**1**), showing (*a*) the dimer and the hydro­gen bonds within the dimeric unit, and (*b*) the stacked aromatic rings and the hydro­gen bonding between four adjacent dimers. Colour code: Li green, C black, O red and H pale pink. In this and later figures where hydro­gen bonds are shown, hydro­gen bonds within the H_3_thba^−^ units are indicated by pink and white striped connections, while other hydro­gen bonds are indicated by black and white connections.

The dimer, Li_2_(H_3_thba)_2_(H_2_O)_8_·2H_2_O (**1**), crystallized from an aqueous solution of LiOH and H_4_thba when the reactants were mixed in stoichiometric ratios ranging from 1:4 to 4:1.The structure of the dimer and the extended packing arrangement are shown in Fig. 5 ▸.

Each octa­hedral Li^+^ ion is bonded to two bridging water mol­ecules [Fig. 5 ▸(*a*)], three terminal water mol­ecules and the 4-hy­droxy group of the H_3_thba^−^ ligand. Hydrogen bonding between the H atom of a hy­droxy group and the O atom of a terminal water mol­ecule coordinated to the adjacent Li centre ‘pinches’ these O atoms together (O⋯O distance ∼2.72 Å).

The H_3_thba^−^ units are closely packed; π–π inter­actions are present between the H_3_thba^−^ ligands, which are arranged in a face-to-face stacking pattern ∼3.50 Å apart, with alternating orientations of the ligand. These anti­parallel stacks separate layers containing Li–O polyhedra. All the complexes of H_3_thba^−^ described in the current work have structures in which layers of metal and O atoms are separated by regions containing closely stacked aromatic rings. This layered architecture is a dominant structural motif in many of the structures reported previously for the alkali salts of H_2_hba and in some other coordination polymers of car­box­yl­ates (Abrahams *et al.*, 2021 ▸; Banerjee & Parise, 2011 ▸).

A remarkable feature of this compound is that each metal centre is bonded to the O atom of a protonated hy­droxy group of the H_3_thba^−^ ligand and water mol­ecules, rather than to the anionic car­box­yl­ate group. As discussed earlier, the car­box­yl­ate group in the H_3_thba^−^ ion is much less basic than most car­box­yl­ate ligands. We suggest that this factor, in combination with the ability of the car­box­yl­ate group to form an extensive hydro­gen-bonded network with lattice water mol­ecules and neighbouring dimers [Fig. 5 ▸(*b*)], results in the preferential binding of metal ions to the hy­droxy groups.

**Figure 6:**
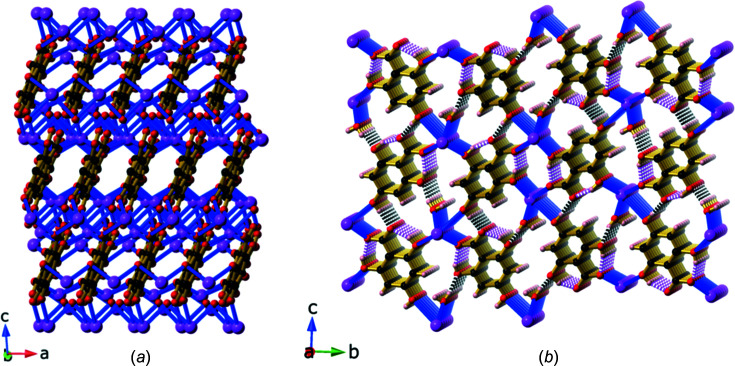
The structure of K(H_3_thba)(H_2_O) (**2**), showing (*a*) a view along the *b* axis highlighting the closely stacked H_3_thba^−^ units between the K–O sheets (H atoms have been omitted for clarity) and (*b*) a view of the face-to-face stacking of the ligands. Colour code: K purple, O red, C black and H pale pink.

We were unable to obtain crystals from the reaction of NaOH and H_4_thba that were suitable for structural analysis. The combination of KOH and H_4_thba yielded crystals of compound **2**, K(H_3_thba)(H_2_O). Like the lithium salt, **2** is also composed of sheets containing metal ions and O atoms that are separated by stacks of aromatic rings [Fig. 6 ▸(*a*)]. However, unlike **1**, the potassium salt forms a three-dimensional ionic network. Each metal centre is six-coordinated and bonded to four H_3_thba^−^ ions. As shown in Fig. 6 ▸(*b*), these anions are closely stacked in a face-to-face parallel pattern along the direction of the *a* axis, which is 3.7740 (4) Å in length. The potassium ions in the layers are spaced this same distance apart and bridged by a combination of water mol­ecules, mono­den­tate car­box­yl­ate groups and hy­droxy groups. Hydrogen bonds link water mol­ecules bonded to one metal centre with hy­droxy groups of ligands bonded to metal centres in adjacent layers.

**Figure 7:**
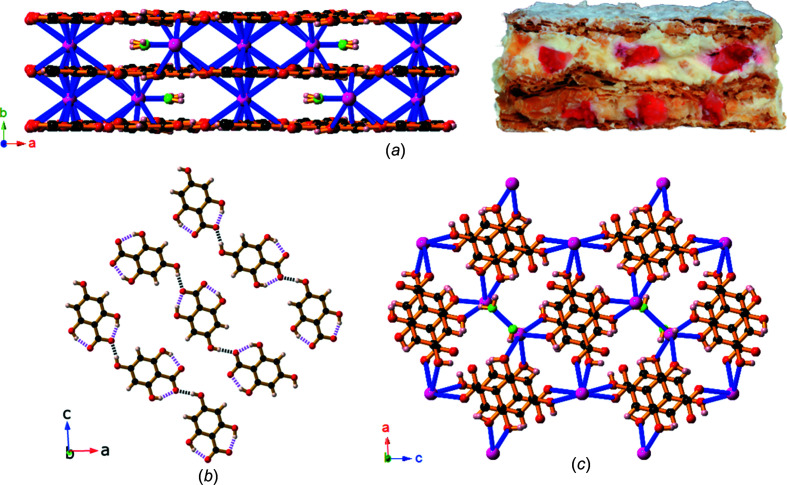
The structure of Rb_2_(H_3_thba)_2_(H_2_O) (**3**) (*a*) in a view down the *c* axis, showing the inter­leaved layers of anions and metal centres that resemble a millefeuille pastry (right), (*b*) with H_3_thba^−^ units forming a plane containing chains linked by hydro­gen bonding and (*c*) with the H_3_thba^−^ units stacked in an anti­parallel face-to-face manner in the direction of the *b* axis. Colour code: Rb purple, car­box­yl­ate and hy­droxy O red, water O green, C black and H pale pink.

Reaction of RbOH with H_4_thba in a 1:1 mixture yielded compound **3**, Rb_2_(H_3_thba)_2_(H_2_O). This compound forms a three-dimensional network, in which layers of H_3_thba^−^ anions are inter­leaved with layers of Rb^+^ ions and water mol­ecules [Fig. 7 ▸(*a*)]. When viewed along the *c* axis, the structure resembles that of the classic French millefeuille pastry, with the ligand layers playing the role of the pastry and metal ions as the filling. The anions form hydro­gen-bonded chains, as shown in Fig. 7 ▸(*b*), and are arranged in anti­parallel stacks ∼3.50 Å apart down the *b* axis [Fig. 7 ▸(*c*)].

Two of the metal centres in the asymmetric unit, Rb1 and Rb2, are 3.45267 (3) Å apart (half the length of the *b* axis) and are arranged in columns within the network. This distance is smaller than the shortest reported Rb⋯Rb distance of 3.5721 (4) Å listed for the structure with refcode TEKXEP in the CSD (Li *et al.*, 2017 ▸; Version 5.43, March 2022 release; Groom *et al.*, 2016 ▸), in which Rb^+^ ions are bridged by O atoms. The third Rb^+^ ion (Rb3) is bonded to a water mol­ecule, and both are located between the H_3_thba^−^ layers, as shown in Fig. 7 ▸(*a*).

**Figure 8:**
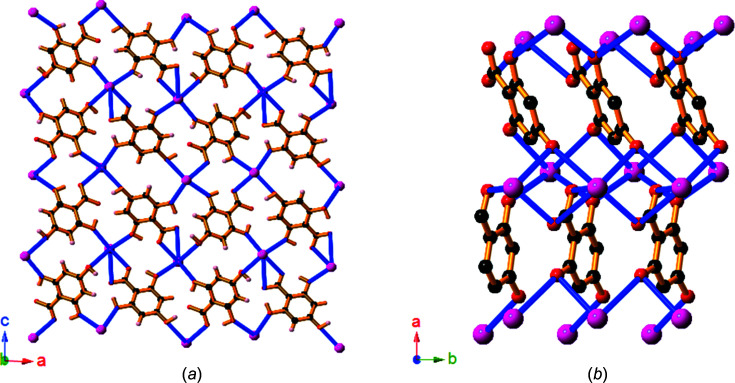
The structure of Cs(H_3_thba) (**4**), showing (*a*) a view along the *b* axis, with the seven- and nine-coordinate Cs^+^ ions and H_3_thba^−^ ions visible, and (*b*) rows of Cs^+^ ions connected by stacks of two different types of H_3_thba^−^ units (H atoms have been omitted for clarity). The metal ions are less than 4.00 Å apart. Colour code: Cs purple, O red, C black and H pale pink.

Compound **4**, Cs(H_3_thba), was isolated from a 1:1 mixture of CsOH and H_4_thba and is a three-dimensional network of Cs^+^ ions and H_3_thba^−^. All hy­droxy groups are bonded to metal ions [Fig. 8 ▸(*a*)]. There are two inequivalent metal ions in the asymmetric unit, one bonded to seven O atoms and the other to nine O atoms, and two different H_3_thba^−^ anions, one in which the car­box­yl­ate group is mono­den­tate with the other bidentate. The H_3_thba^−^ units are closely stacked in a parallel face-to-face fashion [Fig. 8 ▸(*b*)] along the direction of the *b* axis, which is 3.9988 (1) Å in length.

**Figure 9:**
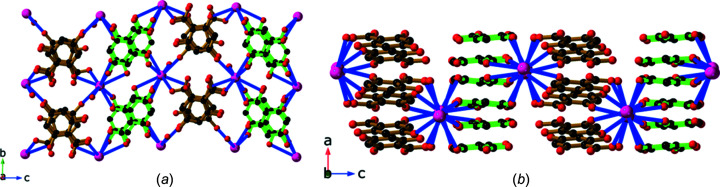
The structure of Cs(H_3_thba)(H_4_thba)(H_2_O) (**5**), showing (*a*) a view along the *a* axis with the separate stacks of H_4_thba (green bonds) and H_3_thba^−^ (brown bonds), and (*b*) the metal centres and closely stacked ligand units viewed down the *b* axis. H atoms have been omitted for clarity. Colour code: Cs purple, O red and C black.

A 1:2 mixture of CsOH and H_4_thba reacts to form compound **5**, Cs(H_3_thba)(H_4_thba)(H_2_O), which contains both neutral H_4_thba and the monoanion, H_3_thba^−^, in a three-dimensional network. The metal centres are eight-coordinate and bonded to six ligands, with the car­box­yl group of the H_4_thba acting in a bidentate mode. The H_4_thba and H_3_thba^−^ units form separate stacks [Fig. 9 ▸(*a*)]. The H_4_thba units are aligned in an anti­parallel fashion, whereas the aromatic rings in the H_3_thba^−^ stacks are rotated relative to each other. Fig. 9 ▸(*b*) shows the stacks of H_4_thba and H_3_thba^−^, and layers of Cs^+^ ions, viewed along the *b* axis. The metal centres are 6.9742 (2) Å apart, which corresponds to the length of the *a* axis.

**Figure 10:**
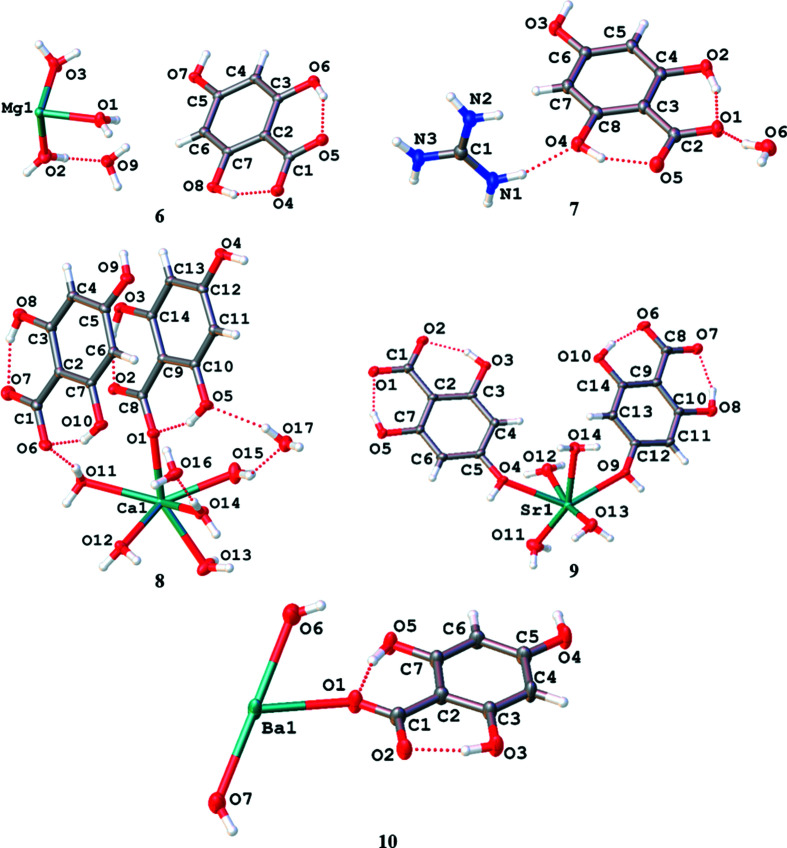
The asymmetric units of [Mg(H_2_O)_6_][H_3_thba]_2_·2H_2_O, **6**, [C(NH_2_)_3_][H_3_thba]·H_2_O, **7**, [Ca_2_(H_2_O)_10_(H_3_thba)_2_][H_3_thba]_2_·4H_2_O, **8**, Sr(H_3_thba)_2_(H_2_O)_4_, **9**, and Ba(H_3_thba)_2_(H_2_O)_4_, **10**, showing the atom-labelling scheme for the compounds. In the case of **6**, only one configuration of the disordered water H atoms is shown for clarity.

With regard to the Group 2 metals, the structures of the asymmetric units of the magnesium, calcium, strontium and barium salts of H_4_thba are shown in Fig. 10 ▸. The structure of the guanidinium salt of H_4_thba is also included to allow comparison with that of the magnesium salt.

The structure of compound **6**, [Mg(H_2_O)_6_][H_3_thba]_2_·2H_2_O, is markedly different to the structures of the other metal salts described previously in this article as the metal centres are not bonded to the organic anions. Instead, the Mg^2+^ ions are present as octa­hedral Mg(H_2_O)_6_^2+^ units; this is unsurprising as the Mg(H_2_O)_6_^2+^ unit is often observed in magnesium compounds (Parsekar *et al.*, 2022 ▸), including in the salt of H_2_hba, [Mg(H_2_O)_6_][Hhba]_2_·2H_2_O (Shnulin *et al.*, 1981 ▸).

**Figure 11:**
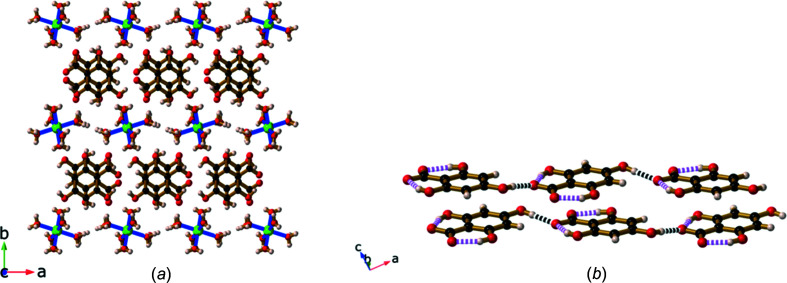
The structure of [Mg(H_2_O)_6_][H_3_thba]_2_·2H_2_O (**6**), showing (*a*) a view down the *c* axis with the separate regions of Mg(H_2_O)_6_^2+^ and H_3_thba^−^ units, and (*b*) hydro­gen bonding between two layers of H_3_thba^−^ units. Colour code: Mg green, O red, C black and H pale pink.

The metal centres are 7.0253 (1) Å apart, which corresponds to the length of the *c* axis. The H_3_thba^−^ units are arranged in stacks with the face-to-face aromatic rings in alternating orientations [Fig. 11 ▸(*a*)], displaying a centroid-to-centroid distance of ∼3.6 Å. Hydrogen bonding links the H_3_thba^−^ units into chains, similar to those seen in **3** [Fig. 11 ▸(*b*)], and there is an extensive hydro­gen-bonding network involving the ligand and the water mol­ecules.

**Figure 12:**
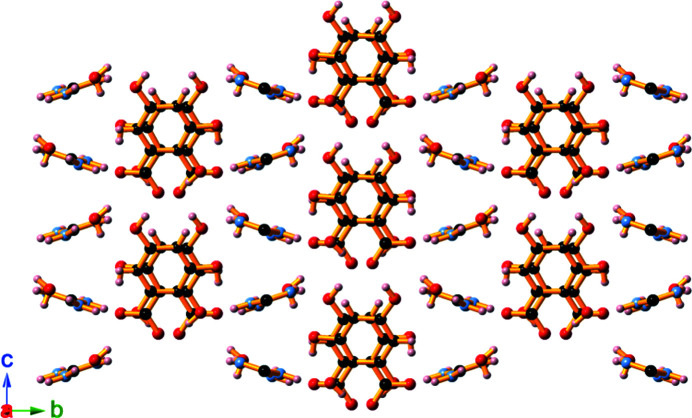
The packing arrangement of [C(NH_2_)_3_][H_3_thba]·H_2_O (**7**). Colour code: N blue, O red, C black and H pale pink.

It is inter­esting to note that the structure of the guanidinium salt, [C(NH_2_)_3_][H_3_thba]·H_2_O (**7**), resembles that of magnesium salt **6** (Fig. 12 ▸) with respect to the relative positions of the cations and the anions. The crystals of both compounds have similar unit-cell dimensions, although **6** has a primitive space group (*P*2_1_/*c*), while **7** is body centred (*Ia*). The orientations of the H_3_thba^−^ units within stacks differ in the two compounds.

**Figure 13:**
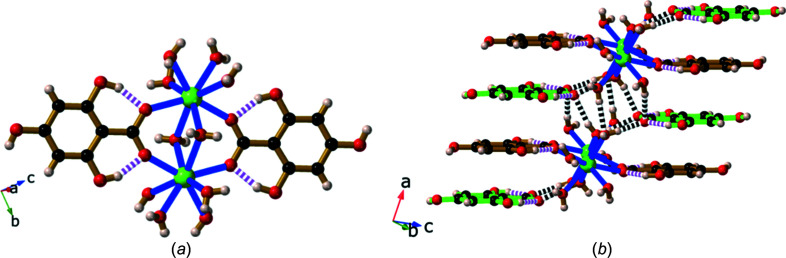
The structure of [Ca_2_(H_3_thba)_2_(H_2_O)_10_][H_3_thba]_2_·4H_2_O (**8**), showing (*a*) the [Ca_2_(H_3_thba)_2_(H_2_O)_10_]^2+^ dimer with the car­box­yl­ate group of the H_3_thba^−^ anion acting in a bridging bidentate mode and (*b*) inter­leaved coordinated H_3_thba^−^ anions (brown bonds) and uncoordinated anions (green bonds) arranged to form stacks. Colour code: Ca green, O red, C black and H pale pink.

The calcium salt of H_4_thba, [Ca_2_(H_3_thba)_2_(H_2_O)_10_][H_3_thba]_2_·4H_2_O (**8**), contains the cationic dimer [Ca_2_(H_3_thba)_2_(H_2_O)_10_]^2+^ [Fig. 13 ▸(*a*)]. Earlier, it was noted that lithium formed an uncharged dimer, Li_2_(H_3_thba)_2_(H_2_O)_8_·2H_2_O (**1**), that is also bridged by two water mol­ecules and two H_3_thba^−^ ligands. In the lithium dimer, the O atom of the 4-hy­droxy group bridges the metal centres, whereas in the calcium dimer, the orientation of the ligand is reversed and the larger and more highly charged Ca^2+^ ions are bridged by anionic car­box­yl­ate groups.

Uncoordinated H_3_thba^−^ anions are inter­leaved between the coordinated anions to form an extended structure in which there are stacks of closely packed ligands [Fig. 13 ▸(*b*)], with hydro­gen bonds between the anions, lattice water mol­ecules and coordinated water mol­ecules.

**Figure 14:**
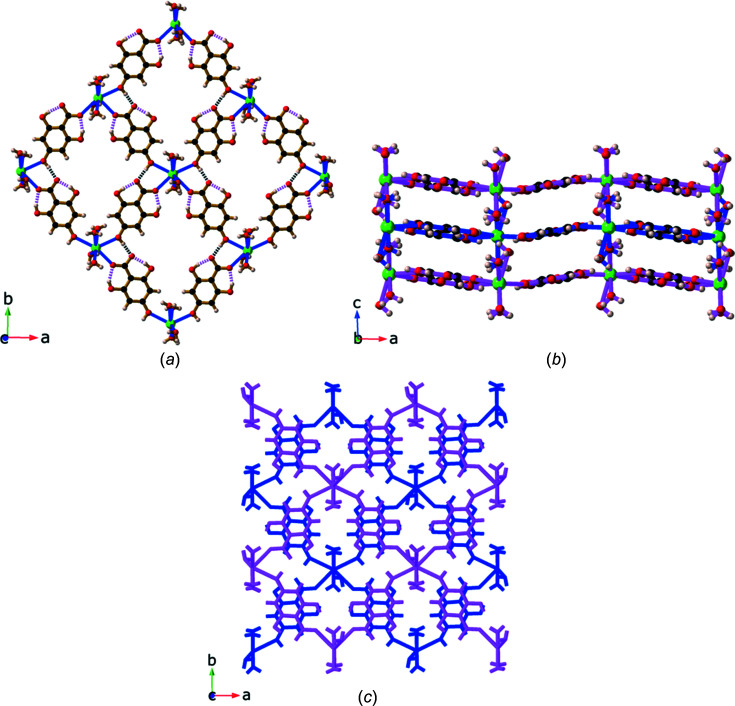
The structure of Sr(H_3_thba)_2_(H_2_O)_4_ (**9**), showing (*a*) a single layer with a 4,4-network structure, (*b*) three of the undulating layers viewed along the *b* axis, with bonds in the layers coloured pink and blue alternately, and (*c*) a stick representation of two layers viewed down the *c* axis, showing the orientation of the sheets relative to each other. Colour code: Sr green, O red, C black and H pale pink.

Compound **9**, Sr(H_3_thba)_2_(H_2_O)_4_, has a beautifully sym­metric two-dimensional 4,4-network architecture (Fig. 14 ▸), formed by coordination of the car­box­yl­ate group at one end of the H_3_thba^−^ ligand and the O atom of the 4-hy­droxy group at the other. As in the other compounds, the H atom on the 4-hy­droxy group participates in hydro­gen bonding to adjacent car­box­yl­ate groups in other ligands. The structure resembles the recently published structure of Mg(Hhba)_2_(H_2_O)_2_·(1,4-dioxane) (Abrahams *et al.*, 2022 ▸), in which there are stacks of parallel networks with the 4,4-topology.

**Figure 15:**
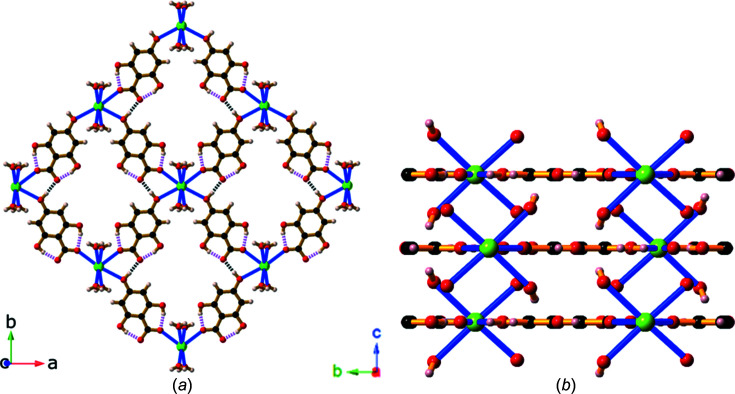
The structure of Ba(H_3_thba)_2_(H_2_O)_4_ (**10**), showing (*a*) a single layer with a 4,4-network structure and (*b*) three layers viewed along the *a* axis. Colour code: Ba green, O red, C black and H pale pink.

As with compound **9**, Ba(H_3_thba)_2_(H_2_O)_4_ (**10**) also has a two-dimensional 4,4-network structure (Fig. 15 ▸), although crystals of the barium compound adopt the ortho­rhom­bic space group *Cmcm*, whereas the strontium compound is monoclinic with the space group *P*2_1_/*c*. A significant difference between the two structures is that the 4,4-network in **9** is undulating, whereas in **10** the network is planar, and these structural differences are presumably the reason for the different space groups.

This description of the metal salts of H_4_thba that contain ionic bonds between the metal centre and H_3_thba^−^ anions concludes with the cerium(III) salt which is of inter­est because the metal is a lanthanide and it is the only compound described in this work that contains 3+ charged metal centres.

**Figure 16:**
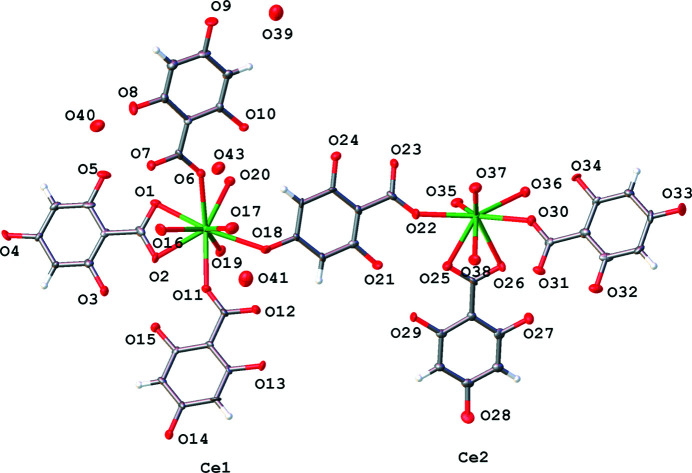
The asymmetric unit of Ce(H_3_thba)_3_(H_2_O)_4_·2H_2_O (**11**), showing the atom-labelling scheme. H atoms bonded to O atoms have not been modelled. For clarity, the labels of C and H atoms have been omitted and only the major disordered component is shown.

**Figure 17:**
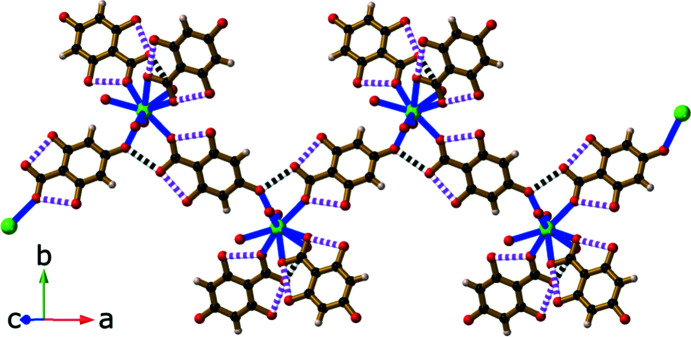
The structure of **11**, showing the zigzag chain formed from Ce^3+^ ions and H_3_thba^−^ units. Colour code: Ce green, O red, C black and H pale pink.

Initial determination of the unit cell of the cerium(III) salt of H_4_thba indicated a *b* axis of ∼9.11 Å; however, upon processing of the reflection data, it became apparent that there was a weak set of reflections consistent with a larger unit cell having *b* = 18.2237 (5) Å. Whilst it was possible to solve and refine the structure with the smaller cell, the use of the larger cell yielded a significantly improved model. The crystal had relatively high mosaicity and exhibited substantial disorder. Nevertheless, the overall structure of the asymmetric unit of the cerium(III) salt of H_4_thba, Ce(H_3_thba)_3_(H_2_O)_4_·2H_2_O (**11**), is clearly resolved and is shown in Fig. 16 ▸. The salt consists of zigzag chains of H_3_thba^−^ anions bonded to nine-coordinate Ce^3+^ ions (Fig. 17 ▸). Each metal centre is bonded to four anions (one bidentate and three mono­den­tate) and four water mol­ecules. The ligands and water mol­ecules in the crystal are disordered over two positions. Although not all H atoms have been identified, the proximity of the O atoms indicates extensive intra- and inter­chain hydro­gen bonding. Zigzag chains extending in the *a* direction form hydro­gen bonds with neighbouring anti­parallel chains to form layers that extend in the *ab* plane. These layers stack along the *c* direction, with close face-to-face contacts and there are uncoordinated water mol­ecules located between the layers.

### Structure description of complexes with coordinate bonds

**Figure 18:**
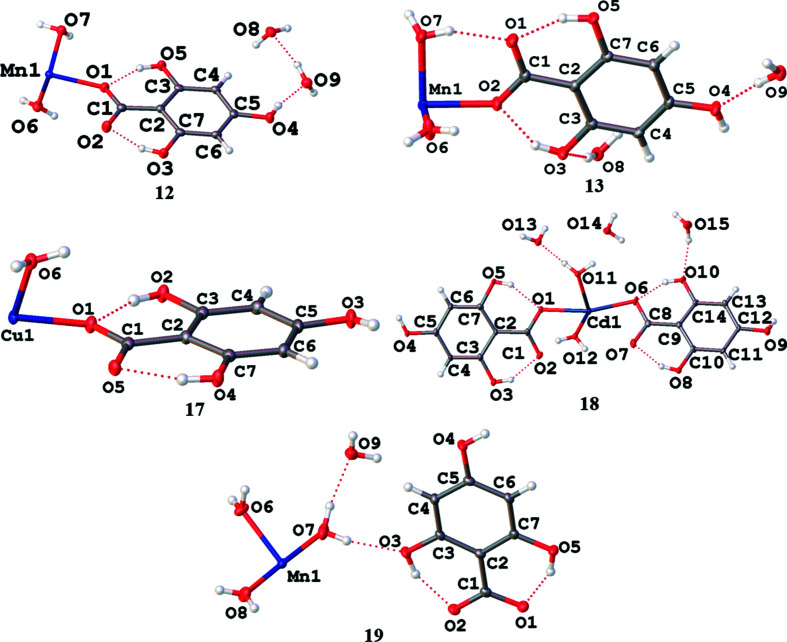
The asymmetric units of Mn(H_3_thba)_2_(H_2_O)_4_·4H_2_O (monoclinic form), **12**, Mn(H_3_thba)_2_(H_2_O)_4_·4H_2_O (triclinic form), **13**, Cu(H_3_thba)_2_(H_2_O)_2_, **17**, Cd(H_3_thba)_2_(H_2_O)_2_·5H_2_O, **18**, and [Mn(H_2_O)_6_][THBA]_2_·2H_2_O, **19**. Compounds Co(H_2_O)_2_(H_3_thba)_2_, **14**, Ni(H_2_O)_2_(H_3_thba)_2_, **15**, and Zn(H_2_O)_2_(H_3_thba)_2_, **16**, are isostructural with **12** and have the same atom-labelling system. In the case of **19**, some of the H atoms in the water mol­ecules are disordered and only one configuration is shown for clarity.

The combination of H_4_thba with divalent *d*-block metal acetates yields a variety of complexes containing H_3_thba^−^. The structures of the asymmetric units of *d*-block metal complexes of H_3_thba^−^ are shown in Fig. 18 ▸.

**Figure 19:**
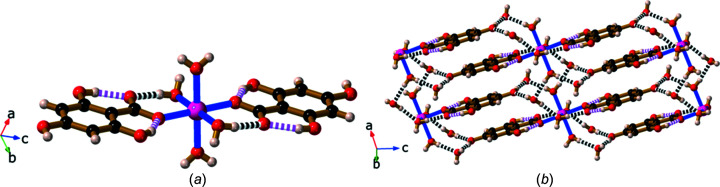
The structure of Mn(H_3_thba)_2_(H_2_O)_4_·4H_2_O (**12**), showing (*a*) the octa­hedral arrangement of atoms around the Mn centre and (*b*) the closely packed alternate stacking of H_3_thba^−^ ligands. Colour code: Mn purple, O red, C black and H pale pink.

Monoclinic crystals of Mn(H_3_thba)_2_(H_2_O)_4_ **·**4H_2_O (**12**) were obtained by heating an aqueous 1:1 mixture of H_4_thba and manganese acetate and leaving the solution to cool, whilst the solvent was allowed to evaporate. The structure of **12** is shown in Fig. 19 ▸. The car­box­yl­ate group of the H_3_thba^−^ ligand binds in a mono­den­tate mode in the *syn* configuration [Mn—O—C—O torsion angle = 9.9 (2)°]. The uncoordinated O atom forms a strong hydro­gen bond (O⋯O distance ∼2.63 Å), with a coordinated water mol­ecule [Fig. 19 ▸(*a*)]. As seen in Fig. 19 ▸(*b*), the H_3_thba^−^ units are closely stacked in alternating orientations in the extended structure (centroid-to-centroid distance ∼3.4 Å).

Triclinic crystals (compound **13**) were also obtained from the same synthesis. Crystals of **13** have the same formula as **12** and indeed a similar mol­ecular structure is obtained for these polymorphs.

Compound **12** is isostructural with the complexes formed when the acetates of cobalt, nickel and zinc react with H_4_thba. These complexes have the formulae Co(H_3_thba)_2_(H_2_O)_4_·4H_2_O (**14**), Ni(H_3_thba)_2_(H_2_O)_4_·4H_2_O (**15**) and Zn(H_3_thba)_2_(H_2_O)_4_·4H_2_O (**16**).

**Figure 20:**
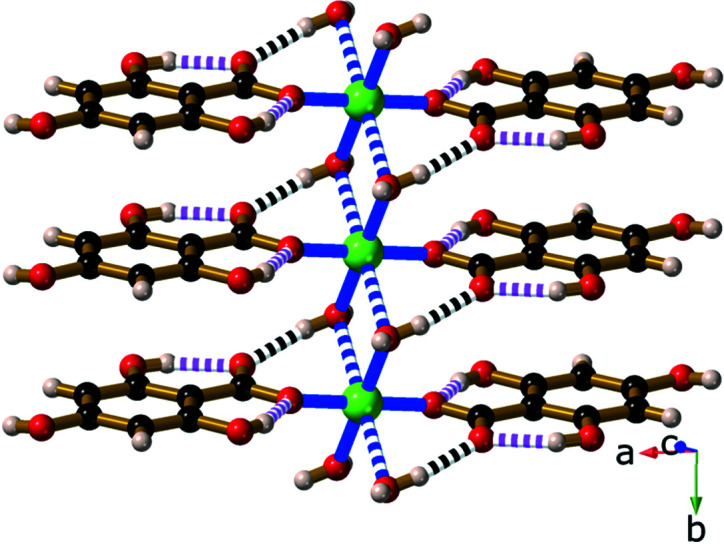
The structure of Cu(H_3_thba)_2_(H_2_O)_2_ (**17**). The copper centre is six-coordinated. A Jahn–Teller distortion is observed in the axial bonds to coordinated water mol­ecules (the blue and white connections). Colour code: Cu green, O red, C black and H pale pink.

In the complex Cu(H_3_thba)_2_(H_2_O)_2_ (**17**), the Cu^II^ centre adopts a tetra­gonally distorted octa­hedral geometry formed by two *trans* mono­den­tate H_3_thba^−^ ligands and four water mol­ecules, each of which is bridging to an adjacent Cu^II^ centre. This results in a chain that extends in the *b* direction, as depicted in Fig. 20 ▸. The organic ligands are bonded to the metal centres in the *syn* configuration [Cu—O—C—O torsion angle = 12.3 (3)°]. Bridging water mol­ecules also participate in hydro­gen bonds with noncoordinated car­box­yl­ate O atoms. The chains are held together by hydro­gen bonds between *ortho*-hy­droxy H atoms and the O atoms of coordinated water mol­ecules in adjacent chains.

**Figure 21:**
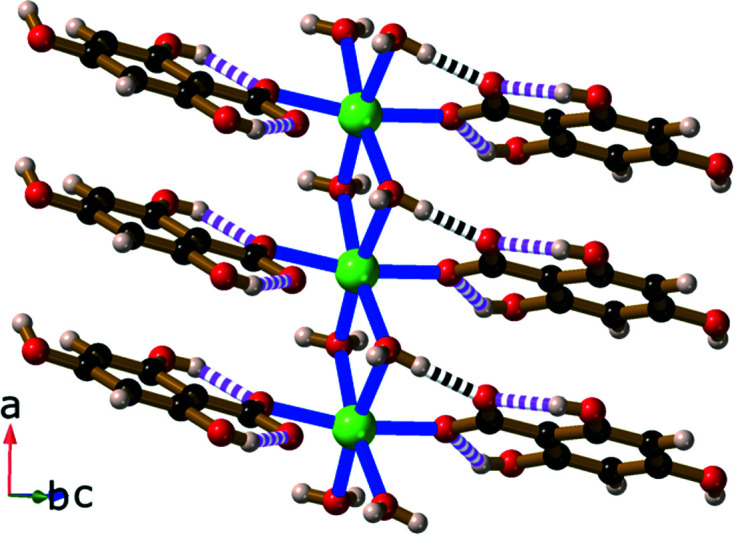
The structure of Cd(H_3_thba)_2_(H_2_O)_2_·3H_2_O (**18**). The structure is similar to **17**, but one of the bridging waters is not involved in hydro­gen bonding. The H_3_thba^−^ units are closely stacked (centroid-to-centroid distance ∼3.6 Å). Colour code: Cd green, O red, C black and H pale pink.

The final *d*-block metal complex described here is Cd(H_3_thba)_2_(H_2_O)_2_·3H_2_O (**18**). Like **17**, it forms chains, but only one of the bridging water mol­ecules participates in intra­chain hydro­gen bonding (Fig. 21 ▸). The chains are held together by hydro­gen bonds between hy­droxy H atoms and both lattice and coordinated water mol­ecules. The H_3_thba^−^ units are bonded to the metal centres in the *syn* configuration [Cd—O—C—O torsion angle = −15.2 (7)°].

Inspection of Fig. 21 ▸ reveals a helical character along the *a* direction. Within the crystal, for which the space group is *P*2_1_2_1_2_1_, all the chains have the same handedness.

It is noted that the same reaction mixture that yielded **12** and **13** produced crystals of a manganese complex with a different structure: [Mn(H_2_O)_6_][H_3_thba]_2_·2H_2_O (**19**). The structure of **19** contains uncoordinated H_3_thba^−^ ions and is very similar to that of magnesium complex **6** discussed earlier.

**Figure 22:**
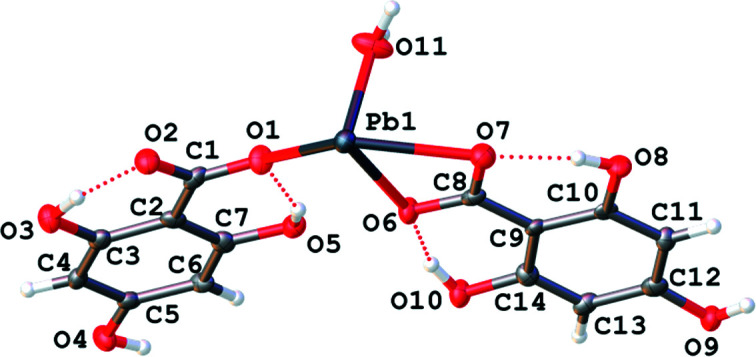
The mol­ecular and asymmetric unit of Pb(H_3_thba)_2_(H_2_O) (**20**), showing the atom-labelling scheme.

**Figure 23:**
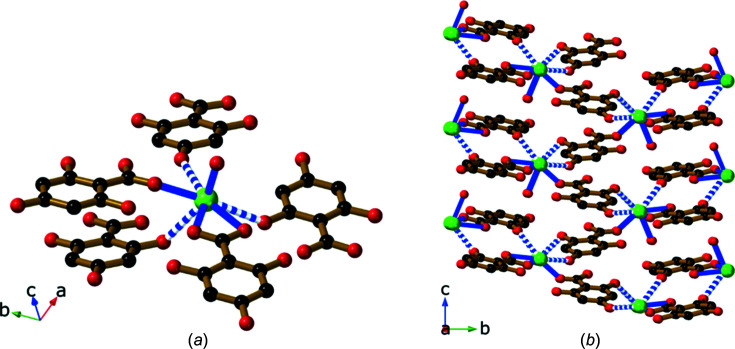
The structure of Pb(H_3_thba)_2_(H_2_O) (**20**), showing (*a*) bonding around the lead centre (noncovalent inter­actions are indicated by blue and white connections) and (*b*) the stacks of H_3_thba^−^ units, viewed down the *a* axis, held together by π–π inter­actions. Hydrogen bonds have been omitted for clarity. Colour code: Pb green, O red, C black and H pale pink.

The final metal-based structure in this section is from the *p*-block and, once again, involves the monoanionic ligand, H_3_thba^−^. Pb(H_3_thba)_2_(H_2_O) (**20**) adopts a discrete monomeric structure, as indicated in Fig. 22 ▸. Each Pb^2+^ ion is four-coordinate and bound to a mono­den­tate H_3_thba^−^ ligand, a bidentate H_3_thba^−^ ligand and a water mol­ecule. Lead com­plexes are linked through hydro­gen bonding. As seen in Figs. 23 ▸(*a*) and 23 ▸(*b*), the H_3_thba^−^ units are stacked along the direction of the *c* axis (centroid-to-centroid distance of the rings of the mono­den­tate ligand ∼3.6 Å).

The Pb^II^ centre exhibits a hemi­directed coordination geometry with all the covalent bonds in one hemisphere of the coordination sphere. The pronounced coordination gap in the Pb^II^ ion created by its lone pair allows the ion to participate in noncovalent inter­actions, known as tetrel bonds (Bauzá *et al.*, 2019 ▸), to O atoms of three adjacent hy­droxy groups [Fig. 23 ▸(*b*); Pb⋯O distances of ∼2.78, ∼2.87 and ∼3.01 Å]. These inter­actions are shorter than the sums of the van der Waals radii but larger than the sums of the covalent radii. The hydro­gen bonds between the mol­ecules, together with the noncovalent bonds and π–π stacking inter­actions between the aromatic rings, link the mol­ecules to create a three-dimensional network.

### Stability of H_4_thba

Whereas previous investigations of complexes of H_2_hba found the ligand to be relatively robust, the deca­rboxylation of H_4_thba to form benzene-1,3,5-triol (phloroglucinol) is a well-known reaction that readily occurs under certain conditions (Schubert & Gardner, 1953 ▸; Zenkevich *et al.*, 2007 ▸). Crystals of the hydrate of benzene-1,3,5-triol, C_6_H_6_O_3_·2H_2_O (Wallwork & Powell, 1957 ▸), were isolated from several reaction mixtures, particularly those that were either heated for extended periods or at temperatures above 50 °C.

**Figure 24:**
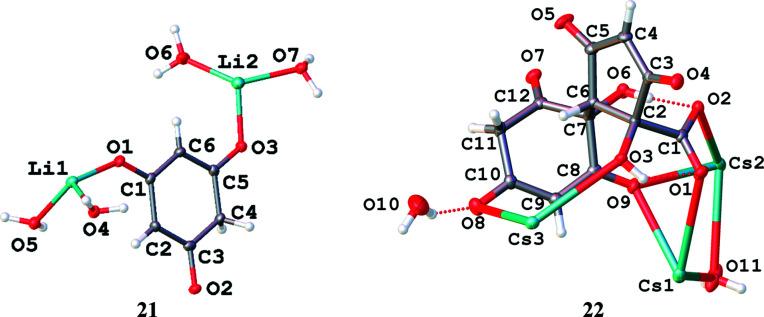
The asymmetric units of Li_2_(C_6_H_4_O_3_)(H_2_O)_4_, **21**, and Cs_3_(C_12_H_7_O_9_)(H_2_O)·H_2_O, **22**, showing the atom-labelling schemes for the compounds. For clarity, only one of the configurations of the H atoms on a disordered O atom (O10) in **22** is shown.

Two new networks composed of decomposition products of H_4_thba and metal ions were also identified. The structures of their asymmetric units are shown in Fig. 24 ▸.

**Figure 25:**
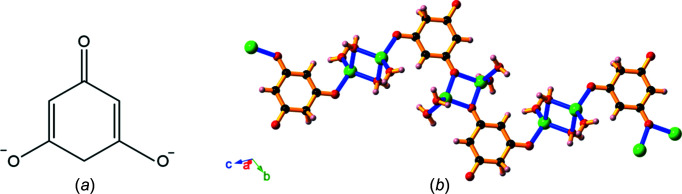
Li_2_(C_6_H_4_O_3_)(H_2_O)_4_ (**21**) is formed by heating a 4:1 reaction mixture of LiOH and H_4_thba. (*a*) The structure of C_6_H_4_O_3_^2−^ and (*b*) chains containing pairs of Li centres bridged by both dianions and water mol­ecules. Colour code: Li green, O red, C black and H pale pink.

Heating a 4:1 mixture of LiOH and H_4_thba in aqueous solution caused deca­rboxylation of H_4_thba and the formation of a π-conjugated dianion with the formula C_6_H_4_O_3_^2−^, shown in Fig. 25 ▸(*a*). This is the keto-alicyclic form of the dianion of phloroglucinol (Highet & Batterham, 1964 ▸). Pairs of Li^+^ ions are bridged by both the dianions and the water mol­ecules to form chains [Fig. 25 ▸(*b*)] of formula Li_2_(C_6_H_4_O_3_)(H_2_O)_4_ (**21**). The uncoordinated O atom of the dianion participates in hydro­gen bonding with adjacent coordinated water mol­ecules. Extensive hydro­gen bonding exists within and between the chains. Of particular inter­est is the noncoordinated O atom of the dianion, which acts as a hydro­gen-bond acceptor from four water mol­ecules.

**Figure 26:**
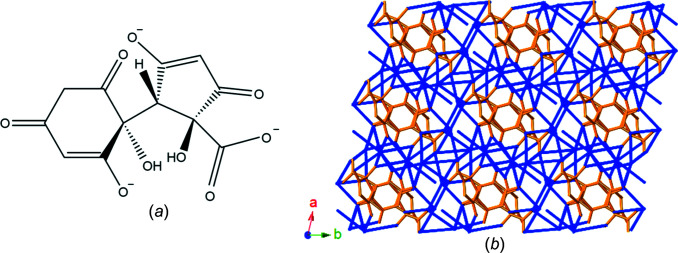
Cs_3_(C_12_H_7_O_9_)(H_2_O)·0.75H_2_O (**22**) is formed by heating a 4:1 aqueous mixture of CsOH and H_4_thba. (*a*) The structure of the C_12_H_7_O_9_^3−^ trianion. (*b*) A stick representation of the view down the *c* axis of the three-dimensional network. H atoms have been omitted. Colour code: Cs—O bonds blue and C—C and C—O bonds orange.

A 4:1 reaction mixture of CsOH and H_4_thba produced a caesium network containing the chiral trianion, C_12_H_7_O_9_^3−^ [Fig. 26 ▸(*a*)], which is comprised of both five- and six-membered rings, and two OCCCO π-systems. The trianion combines with Cs^+^ ions to form Cs_3_(C_12_H_7_O_9_)(H_2_O)·0.75H_2_O (**22**), an intricate three-dimensional network in which the organic anion inter­acts with numerous caesium centres. The O atom of the solvent water mol­ecule has 75% occupancy based upon refinement of the site occupancy. The organic anion is chiral and the crystal consists of a racemic mixture of anions. No further characterization of the anion was performed. The structure is shown in stick representation in Fig. 26 ▸(*b*).

### Discussion of structural trends

This investigation has shown that there is a wide variation in the structures of the crystalline compounds formed by the H_3_thba^−^ ion when combined with metal ions in aqueous solutions. Close face-to-face packing of the aromatic rings is apparent in many of the structures, leading to layers of metal–oxygen polyhedra separated by organic groups.

In compounds of the *s*-block metals, where the inter­actions of the metal centres with the car­box­yl­ate O atoms are mainly ionic and the directionality of bonds is of less importance, the anion binds to up to three metal centres *via* several of the car­box­yl­ate binding modes shown in Fig. 1 ▸, *viz.* modes I (compounds **5**, **9** and **10**), II (**5**), III (**8**), IV (**2** and **3**) and V (**4**).

In compounds where the metal centre is more likely to form coordinate bonds, the H_3_thba^−^ ion exhibits far less variation in its binding modes. When compared to simple aromatic car­box­yl­ate ligands, including the anions Hhba^−^ and hba^2−^, there is less variety in the coordination modes involving coordinate bonds to metal centres. Whereas other ligands bond readily to two, three or four transition-metal centres, most of the compounds containing coordination bonds described in this investigation have the car­box­yl­ate groups acting solely in a mono­den­tate mode (binding mode I), inter­acting with just one metal ion in the forward, or *syn*, direction (compounds **12**–**18**). The Pb complex (**20**) is an exception, with one ligand mono­den­tate and the other forming a four-membered chelate.

The relatively low basicity of the H_3_thba^−^ anion appears to be a dominant factor in the nature of the complexes it forms, making it less likely to inter­act with multiple metal centres. The location of the *ortho*-hy­droxy groups and intra­molecular hydro­gen bonds also appears to prevent the car­box­yl­ate group from associating with metal ions in an *anti* configuration.

It is noteworthy that in the ionic salts described, the atoms of the car­box­yl­ate groups are in, or close to being in, the plane of the aromatic ring. Earlier studies of the alkali metal salts of H_2_hba (Abrahams *et al.*, 2021 ▸) found pronounced rotation of the atoms in the car­box­yl­ate groups away from the plane of the ring in *M*(Hhba)·H_2_O compounds (*M* = K, 25.1°; Rb, 26.9°; Cs, 24.5°), presumably as a result of crystal packing forces and other steric considerations. In the case of the H_3_thba^−^ ion, however, the hydro­gen bonds between the car­box­yl­ate group and the H atoms of the *ortho*-hy­droxy groups appear to constrain the entire metal–car­box­yl­ate–aromatic ring system to a planar conformation.

The dimers formed by lithium and calcium with H_3_thba^−^ (**1** and **8**) provide a contrast with respect to preferred coordination modes. In each case, a pair of H_3_thba^−^ units bridge the metal ions; however, in the calcium dimer, it is the car­box­yl­ate group of each ligand that spans the metal centres, whereas in the lithium dimer, the O atoms of the 4-hy­droxy groups link a pair of metal centres. This role reversal of the functional groups is likely to reflect the difference in electrostatic attraction between the anions and the 1+ and 2+ charged metal centres, and the ability of the car­box­yl­ate and hy­droxy groups to form hydro­gen bonds with neighbouring water mol­ecules and dimers.

The final trend considered here relates to the metal binding of the hy­droxy groups of H_3_thba^−^. The Group 1 metal ions K^+^, Rb^+^ and Cs^+^ inter­act with all of the O atoms of the hy­droxy groups (compounds **2**–**5**), whereas the Group 2 metal ions Sr^2+^ and Ba^2+^, and also Ce^3+^, restrict their hy­droxy inter­actions to the 4-hy­droxy group (**9**–**11**). In part, this may reflect the presence of a greater number of ligands per metal centre in the salts containing more highly charged cations and, therefore, the greater availability of oxygen donor atoms for bonding. The metals that form traditional coordination bonds did not form bonds to the hy­droxy groups.

## Conclusion

The complexes formed by H_4_thba described in this study display a wide range of inter­esting structures, including discrete monomers, dimers, chains, and two- and three-dimensional networks (and even one that resembles a French pastry). The H_3_thba^−^ ligands in the lattices are closely packed with π–π stacking inter­actions between the aromatic rings. Hydrogen bonds clearly play a key structure-directing role in all compounds considered.

The car­box­yl­ate groups in these complexes are of special inter­est because this group can typically adopt a variety of binding modes. The intra­molecular hydro­gen bonds between the *ortho*-hy­droxy groups and the car­box­yl­ate group in the H_3_thba^−^ ion confer a planar rigid configuration upon the ligand that appears to limit its ability to form bonds, particularly directional coordination bonds. As discussed above, the low basicity of the car­box­yl­ate group in the H_3_thba^−^ anion provides a contrast with the typical coordination behaviour of other car­box­yl­ate anions, resulting in a lower affinity for metal centres. Furthermore, the *ortho*-hy­droxy groups appear to limit the availability of coordination modes that are commonly encountered with other car­box­yl­ate ligands.

The fact that almost all the complexes described in this report contain the monoanion, H_3_thba^−^, leads us to contemplate the use of more strongly basic reaction conditions to synthesize potentially inter­esting frameworks with networks that contain the dianion, trianion or even tetra­anion, possibly using nona­queous solvents for their synthesis. This may prove difficult as harsher reaction conditions may result in the types of decomposition of H_4_thba described in Section 3.3.

This investigation was highly successful in allowing senior secondary school students to experience genuine scientific discovery whilst giving them the opportunity to learn some basic principles of X-ray crystallography. In addition, students were able to appreciate the power of X-ray crystallography in being able to obtain detailed structural information at the mol­ecular level. It was pleasing to see students responding enthusiastically to the opportunity to perform research. Students were keen to experiment, to discover the nature of the new compounds they synthesized and to learn more about the roles of strong and weak bonding inter­actions in the structure of matter.

## Supplementary Material

Crystal structure: contains datablock(s) 1_li_h3thba, 2_KH3thba_cc66b, 3_Rb_H3thba_newrun_large_mask_tw, 4_Cs_H3thba_twin, 5_Cs_H4thba_H3thba_cc_c2thba_2to1, 6_Mg_H3thba_cc_mg_thba_1to1_pl, 7_guanidinium_H3thba_cc124f, 8_Ca_H3thba_cc124c, 9_Sr_H3thba_cc_srthba_twin1_hklf4, 10_Ba_H3thba_gaussian_april2022, 11_Ce_H3thba_weak_peaks_pl, 12_Mn_H3thba_cc124b, 13_Mn_H3THBA_triclinic_cc_mnhthba_5, 14_Co_H3thba_cc120b_2, 15_Ni_H3thba_cc2120c, 16_Zn_H3thba_gaussian_abs.hkl, 17_Cu_H3thba_cc_126d, 18_Cd_H3thba_cc_cdthba, 19_MnH2O6_H3thba_ccmnh2o6thba, 20_Pb_H3thba_cc_pbthba_frompboac_2_autored, 21_Li_C6H4O3_cc_lithba_4to1, 22_Cs_C12H7O9_cc_cs_trihy, global. DOI: 10.1107/S2053229622009901/ep3026sup1.cif

Structure factors: contains datablock(s) 1_li_h3thba. DOI: 10.1107/S2053229622009901/ep30261_li_h3thbasup2.hkl

Structure factors: contains datablock(s) 2_KH3thba_cc66b. DOI: 10.1107/S2053229622009901/ep30262_KH3thba_cc66bsup3.hkl

Structure factors: contains datablock(s) 3_Rb_H3thba_newrun_large_mask_tw. DOI: 10.1107/S2053229622009901/ep30263_Rb_H3thba_newrun_large_mask_twsup4.hkl

Structure factors: contains datablock(s) 4_Cs_H3thba_twin. DOI: 10.1107/S2053229622009901/ep30264_Cs_H3thba_twinsup5.hkl

Structure factors: contains datablock(s) 5_Cs_H4thba_H3thba_cc_c2thba_2to1. DOI: 10.1107/S2053229622009901/ep30265_Cs_H4thba_H3thba_cc_c2thba_2to1sup6.hkl

Structure factors: contains datablock(s) 6_Mg_H3thba_cc_mg_thba_1to1_pl. DOI: 10.1107/S2053229622009901/ep30266_Mg_H3thba_cc_mg_thba_1to1_plsup7.hkl

Structure factors: contains datablock(s) 7_guanidinium_H3thba_cc124f. DOI: 10.1107/S2053229622009901/ep30267_guanidinium_H3thba_cc124fsup8.hkl

Structure factors: contains datablock(s) 8_Ca_H3thba_cc124c. DOI: 10.1107/S2053229622009901/ep30268_Ca_H3thba_cc124csup9.hkl

Structure factors: contains datablock(s) 9_Sr_H3thba_cc_srthba_twin1_hklf4. DOI: 10.1107/S2053229622009901/ep30269_Sr_H3thba_cc_srthba_twin1_hklf4sup10.hkl

Structure factors: contains datablock(s) 10_Ba_H3thba_gaussian_april2022. DOI: 10.1107/S2053229622009901/ep302610_Ba_H3thba_gaussian_april2022sup11.hkl

Structure factors: contains datablock(s) 11_Ce_H3thba_weak_peaks_pl. DOI: 10.1107/S2053229622009901/ep302611_Ce_H3thba_weak_peaks_plsup12.hkl

Structure factors: contains datablock(s) 12_Mn_H3thba_cc124b. DOI: 10.1107/S2053229622009901/ep302612_Mn_H3thba_cc124bsup13.hkl

Structure factors: contains datablock(s) 13_Mn_H3THBA_triclinic_cc_mnhthba_5. DOI: 10.1107/S2053229622009901/ep302613_Mn_H3THBA_triclinic_cc_mnhthba_5sup14.hkl

Structure factors: contains datablock(s) 14_Co_H3thba_cc120b_2. DOI: 10.1107/S2053229622009901/ep302614_Co_H3thba_cc120b_2sup15.hkl

Structure factors: contains datablock(s) 15_Ni_H3thba_cc2120c. DOI: 10.1107/S2053229622009901/ep302615_Ni_H3thba_cc2120csup16.hkl

Structure factors: contains datablock(s) 16_Zn_H3thba_gaussian_abs.hkl. DOI: 10.1107/S2053229622009901/ep302616_Zn_H3thba_gaussian_abs.hklsup17.hkl

Structure factors: contains datablock(s) 17_Cu_H3thba_cc_126d. DOI: 10.1107/S2053229622009901/ep302617_Cu_H3thba_cc_126dsup18.hkl

Structure factors: contains datablock(s) 18_Cd_H3thba_cc_cdthba. DOI: 10.1107/S2053229622009901/ep302618_Cd_H3thba_cc_cdthbasup19.hkl

Structure factors: contains datablock(s) 19_MnH2O6_H3thba_ccmnh2o6thba. DOI: 10.1107/S2053229622009901/ep302619_MnH2O6_H3thba_ccmnh2o6thbasup20.hkl

Structure factors: contains datablock(s) 20_Pb_H3thba_cc_pbthba_frompboac_2_autored. DOI: 10.1107/S2053229622009901/ep302620_Pb_H3thba_cc_pbthba_frompboac_2_autoredsup21.hkl

Structure factors: contains datablock(s) 21_Li_C6H4O3_cc_lithba_4to1. DOI: 10.1107/S2053229622009901/ep302621_Li_C6H4O3_cc_lithba_4to1sup22.hkl

Structure factors: contains datablock(s) 22_Cs_C12H7O9_cc_cs_trihy. DOI: 10.1107/S2053229622009901/ep302622_Cs_C12H7O9_cc_cs_trihysup23.hkl

CCDC references: 2212034, 2212033, 2212032, 2212031, 2212030, 2212029, 2212028, 2212027, 2212026, 2212025, 2212024, 2212023, 2212022, 2212021, 2212020, 2212019, 2212018, 2212017, 2212016, 2212015, 2212014, 2212013

## Figures and Tables

**Table:** Diffraction data were measured using a Rigaku XtalLAB Synergy-S (Dualflex, HyPix) diffractometer, except for the data for compound **22**, for which an Oxford Diffraction Supernova (Dual, Atlas) diffractometer was used. Data were collected at 100 K, except for compounds **12** (103 K) and **20** (107 K). Cu *K*α radiation was employed, with the exception of the data collections for compounds **3**, **4** and **22**, which used Mo *K*α radiation. H atoms were treated by a mixture of independent and constrained refinement, except for compound **11**, for which H-atom parameters were constrained.

	**1**	**2**	**3**	**4**
Crystal data				
Chemical formula	[Li_2_(C_7_H_5_O_5_)_2_(H_2_O)_8_]·2H_2_O	[K(C_7_H_5_O_5_)(H_2_O)]	[Rb_2_(C_7_H_5_O_5_)_2_(H_2_O)]	[Cs(C_7_H_5_O_5_)]
*M*_r_	532.26	226.23	527.18	302.02
Crystal system, space group	Triclinic, *P* 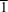	Monoclinic, *P*2_1_/*c*	Monoclinic, *C*2/*c*	Monoclinic, *C*2/*c*
*a*, *b*, *c* (Å)	6.8553 (3), 8.5698 (2), 10.3468 (4)	3.77740 (4), 30.1580 (3), 15.00812 (18)	22.2677 (11), 6.9047 (3), 22.2964 (8)	27.8456 (7), 3.9988 (1), 29.2588 (9)
α, β, γ (°)	95.637 (3), 102.395 (3), 108.297 (3)	90, 94.9465 (10), 90	90, 92.908 (4), 90	90, 92.003 (3), 90
*V* (Å^3^)	554.61 (4)	1703.34 (3)	3423.7 (3)	3255.95 (15)
*Z*	1	8	8	16
μ (mm^−1^)	1.33	5.57	5.78	4.53
Crystal size (mm)	0.34 × 0.21 × 0.10	0.26 × 0.05 × 0.03	0.36 × 0.1 × 0.05	0.37 × 0.18 × 0.07

Data collection				
Absorption correction	Gaussian (*CrysAlis PRO*; Rigaku OD, 2018–2021 ▸)	Gaussian (*CrysAlis PRO*; Rigaku OD, 2018–2021 ▸)	Multi-scan (*CrysAlis PRO*; Rigaku OD, 2018–2021 ▸)	Multi-scan (*CrysAlis PRO*; Rigaku OD, 2018–2021 ▸)
*T*_min_, *T*_max_	0.283, 1.000	0.284, 1.000	0.296, 1.000	0.634, 1.000
No. of measured, independent and observed [*I* > 2σ(*I*)] reflections	5754, 2210, 2042	15764, 3563, 3234	3013, 3013, 2238	4281, 4281, 4166
*R*_int_	0.022	0.048	–	–
(sin θ/λ)_max_ (Å^−1^)	0.631	0.634	0.603	0.602

Refinement				
*R*[*F* ^2^ > 2σ(*F* ^2^)], *wR*(*F* ^2^), *S*	0.032, 0.098, 1.11	0.037, 0.106, 1.04	0.034, 0.096, 1.06	0.054, 0.160, 1.17
No. of reflections	2210	3563	3013	4281
No. of parameters	183	283	258	254
No. of restraints	13	21	15	181
Δρ_max_, Δρ_min_ (e Å^−3^)	0.37, −0.29	0.58, −0.29	0.84, −0.71	1.66, −1.33

**Table:** 

	**5**	**6**	**7**	**8**
Crystal data				
Chemical formula	[Cs(C_7_H_5_O_5_)(C_7_H_6_O_5_)(H_2_O)]	[Mg(H_2_O)_6_](C_7_H_5_O_5_)_2_·2H_2_O	CH_6_N_3_^+^·C_7_H_5_O_5_^−^·H_2_O	[Ca_2_(C_7_H_5_O_5_)_2_(H_2_O)_10_](C_7_H_5_O_5_)_2_·4H_2_O
*M*_r_	490.15	506.66	247.21	1008.82
Crystal system, space group	Orthorhombic, *P* *b* *c* *a*	Monoclinic, *P*2_1_/*c*	Monoclinic, *I* *a*	Triclinic, *P* 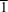
*a*, *b*, *c* (Å)	6.9742 (2), 15.2467 (4), 29.5616 (7)	7.1116 (2), 20.5162 (5), 7.0253 (1)	6.9815 (2), 20.1684 (6), 7.4156 (2)	6.9836 (2), 9.9150 (3), 14.4214 (4)
α, β, γ (°)	90, 90, 90	90, 91.148 (2), 90	90, 91.627 (2), 90	88.420 (2), 86.377 (2), 86.733 (3)
*V* (Å^3^)	3143.39 (14)	1024.81 (4)	1043.74 (5)	994.67 (5)
*Z*	8	2	4	1
μ (mm^−1^)	18.99	1.63	1.18	3.57
Crystal size (mm)	0.16 × 0.13 × 0.05	0.24 × 0.08 × 0.05	0.44 × 0.11 × 0.07	0.52 × 0.10 × 0.05

Data collection				
Absorption correction	Multi-scan (*CrysAlis PRO*; Rigaku OD, 2018–2021 ▸)	Multi-scan (*CrysAlis PRO*; Rigaku OD, 2018–2021 ▸)	Multi-scan (*CrysAlis PRO*; Rigaku OD, 2018–2021 ▸)	Multi-scan (*CrysAlis PRO*; Rigaku OD, 2018–2021 ▸)
*T*_min_, *T*_max_	0.142, 1.000	0.640, 1.000	0.566, 1.000	0.564, 1.000
No. of measured, independent and observed [*I* > 2σ(*I*)] reflections	14256, 3278, 2946	8140, 2071, 1881	3719, 1421, 1383	11872, 4081, 3692
*R*_int_	0.057	0.028	0.045	0.047
(sin θ/λ)_max_ (Å^−1^)	0.635	0.632	0.632	0.633

Refinement				
*R*[*F* ^2^ > 2σ(*F* ^2^)], *wR*(*F* ^2^), *S*	0.042, 0.116, 1.07	0.033, 0.097, 1.06	0.049, 0.138, 1.07	0.039, 0.114, 1.05
No. of reflections	3278	2071	1421	4081
No. of parameters	264	177	181	349
No. of restraints	10	5	17	15
Δρ_max_, Δρ_min_ (e Å^−3^)	1.16, −1.05	0.26, −0.28	0.30, −0.29	0.45, −0.50
Absolute structure	–	–	Flack *x* determined using 305 quotients [(*I* ^+^) − (*I* ^−^)]/[(*I* ^+^) + (*I* ^−^)] (Parsons *et al.*, 2013 ▸)	–
Absolute structure parameter	–	–	0.3 (3)	–

**Table:** 

	**9**	**10**	**11**	**12**
Crystal data				
Chemical formula	[Sr(C_7_H_5_O_5_)_2_(H_2_O)_4_]	[Ba(C_7_H_5_O_5_)_2_(H_2_O)_4_]	[Ce(C_7_H_5_O_5_)_3_(H_2_O)_4_]·2H_2_O	[Mn(C_7_H_5_O_5_)_2_(H_2_O)_4_]·4H_2_O
*M*_r_	497.90	547.62	734.38	537.29
Crystal system, space group	Monoclinic, *P*2_1_/*c*	Orthorhombic, *C* *m* *c* *m*	Monoclinic, *P*2_1_/*n*	Monoclinic, *P*2_1_/*n*
*a*, *b*, *c* (Å)	16.2436 (6), 16.0663 (7), 6.9876 (3)	16.9238 (7), 16.1932 (7), 7.0336 (3)	16.7404 (3), 18.2237 (5), 18.9013 (6)	6.9747 (1), 12.7242 (2), 12.4073 (2)
α, β, γ (°)	90, 92.171 (3), 90	90, 90, 90	90, 114.273 (2), 90	90, 103.102 (2), 90
*V* (Å^3^)	1822.28 (13)	1927.56 (14)	5256.5 (3)	1072.45 (3)
*Z*	4	4	8	2
μ (mm^−1^)	4.84	16.71	14.30	5.85
Crystal size (mm)	0.21 × 0.16 × 0.03	0.11 × 0.08 × 0.03	0.31 × 0.19 × 0.14	0.57 × 0.12 × 0.10

Data collection				
Absorption correction	Multi-scan (*CrysAlis PRO*; Rigaku OD, 2018–2021 ▸)	Gaussian (*CrysAlis PRO*; Rigaku OD, 2018–2021 ▸)	Multi-scan (*CrysAlis PRO*; Rigaku OD, 2018–2021 ▸)	Multi-scan (*CrysAlis PRO*; Rigaku OD, 2018–2021 ▸)
*T*_min_, *T*_max_	0.677, 1.000	0.288, 0.684	0.463, 1.000	0.431, 1.000
No. of measured, independent and observed [*I* > 2σ(*I*)] reflections	6577, 6577, 6185	3778, 947, 921	32351, 9213, 6870	8616, 2248, 2087
*R*_int_	–	0.033	0.054	0.044
(sin θ/λ)_max_ (Å^−1^)	0.635	0.592	0.595	0.634

Refinement				
*R*[*F* ^2^ > 2σ(*F* ^2^)], *wR*(*F* ^2^), *S*	0.069, 0.199, 1.06	0.044, 0.121, 1.11	0.056, 0.171, 1.08	0.032, 0.089, 1.07
No. of reflections	6577	947	9213	2248
No. of parameters	290	101	1034	185
No. of restraints	14	7	398	11
Δρ_max_, Δρ_min_ (e Å^−3^)	2.88, −1.89	3.23, −1.34	2.46, −2.00	0.44, −0.33

**Table:** 

	**13**	**14**	**15**	**16**
Crystal data				
Chemical formula	[Mn(C_7_H_5_O_5_)_2_(H_2_O)_4_]·4H_2_O	[Co(C_7_H_5_O_5_)_2_(H_2_O)_4_]·4H_2_O	[Ni(C_7_H_5_O_5_)_2_(H_2_O)_4_]·4H_2_O	[Zn(C_7_H_5_O_5_)_2_(H_2_O)_4_]·4H_2_O
*M*_r_	537.29	541.28	541.06	547.72
Crystal system, space group	Triclinic, *P* 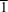	Monoclinic, *P*2_1_/*n*	Monoclinic, *P*2_1_/*n*	Monoclinic, *P*2_1_/*n*
*a*, *b*, *c* (Å)	7.4216 (1), 7.6597 (1), 11.1934 (1)	6.9262 (1), 12.6128 (1), 12.3289 (1)	6.9107 (1), 12.5958 (2), 12.2782 (2)	6.9305 (1), 12.6412 (1), 12.3144 (1)
α, β, γ (°)	100.017 (1), 90.262 (1), 117.689 (2)	90, 102.524 (1), 90	90, 102.279 (1), 90	90, 102.542 (1), 90
*V* (Å^3^)	552.19 (2)	1051.41 (2)	1044.32 (3)	1053.12 (2)
*Z*	1	2	2	2
μ (mm^−1^)	5.68	7.26	2.20	2.48
Crystal size (mm)	0.28 × 0.19 × 0.08	0.25 × 0.21 × 0.16	0.2 × 0.18 × 0.08	0.17 × 0.09 × 0.08

Data collection				
Absorption correction	Multi-scan (*CrysAlis PRO*; Rigaku OD, 2018–2021 ▸)	Multi-scan (*CrysAlis PRO*; Rigaku OD, 2018–2021 ▸)	Multi-scan (*CrysAlis PRO*; Rigaku OD, 2018–2021 ▸)	Gaussian (*CrysAlis PRO*; Rigaku OD, 2018–2021 ▸)
*T*_min_, *T*_max_	0.617, 1.000	0.745, 1.000	0.910, 1.000	0.562, 1.000
No. of measured, independent and observed [*I* > 2σ(*I*)] reflections	6840, 2300, 2295	6725, 2135, 2037	7237, 2073, 1928	6622, 2067, 1975
*R*_int_	0.030	0.018	0.028	0.019
(sin θ/λ)_max_ (Å^−1^)	0.634	0.632	0.631	0.632

Refinement				
*R*[*F* ^2^ > 2σ(*F* ^2^)], *wR*(*F* ^2^), *S*	0.028, 0.081, 1.07	0.025, 0.069, 1.04	0.030, 0.084, 1.04	0.022, 0.061, 1.07
No. of reflections	2300	2135	2073	2067
No. of parameters	185	182	184	185
No. of restraints	13	11	0	11
Δρ_max_, Δρ_min_ (e Å^−3^)	0.40, −0.36	0.33, −0.28	0.38, −0.70	0.39, −0.32

**Table:** 

	**17**	**18**	**19**	**20**
Crystal data				
Chemical formula	[Cu(C_7_H_5_O_5_)_2_(H_2_O)_2_]	[Cd(C_7_H_5_O_5_)_2_(H_2_O)_2_]·3H_2_O	[Mn(H_2_O)_6_](C_7_H_5_O_5_)_2_·2H_2_O	[Pb(C_7_H_5_O_5_)_2_(H_2_O)]
*M*_r_	437.79	540.70	537.29	563.43
Crystal system, space group	Monoclinic, *P*2_1_/*c*	Orthorhombic, *P*2_1_2_1_2_1_	Monoclinic, *P*2_1_/*c*	Monoclinic, *P*2_1_/*c*
*a*, *b*, *c* (Å)	14.2175 (2), 3.5856 (1), 14.4724 (2)	3.61408 (4), 18.51333 (18), 26.7820 (2)	7.0973 (1), 20.6804 (2), 7.0590 (1)	7.47743 (16), 27.8276 (5), 7.12866 (17)
α, β, γ (°)	90, 97.782 (1), 90	90, 90, 90	90, 91.642 (1), 90	90, 90.040 (2), 90
*V* (Å^3^)	730.98 (3)	1791.95 (3)	1035.66 (2)	1483.32 (6)
*Z*	2	4	2	4
μ (mm^−1^)	2.84	10.57	6.05	22.76
Crystal size (mm)	0.39 × 0.06 × 0.02	0.16 × 0.08 × 0.04	0.38 × 0.12 × 0.09	0.19 × 0.04 × 0.02

Data collection				
Absorption correction	Gaussian (*CrysAlis PRO*; Rigaku OD, 2018–2021 ▸)	Multi-scan (*CrysAlis PRO*; Rigaku OD, 2018–2021 ▸)	Gaussian (*CrysAlis PRO*; Rigaku OD, 2018–2021 ▸)	Gaussian (*CrysAlis PRO*; Rigaku OD, 2018–2021 ▸)
*T*_min_, *T*_max_	0.553, 1.000	0.680, 1.000	0.303, 1.000	0.142, 0.871
No. of measured, independent and observed [*I* > 2σ(*I*)] reflections	4609, 1541, 1435	8185, 3452, 3330	12509, 2174, 2073	6087, 2490, 2282
*R*_int_	0.036	0.047	0.042	0.048
(sin θ/λ)_max_ (Å^−1^)	0.634	0.634	0.633	0.595

Refinement				
*R*[*F* ^2^ > 2σ(*F* ^2^)], *wR*(*F* ^2^), *S*	0.037, 0.107, 1.11	0.031, 0.077, 1.03	0.029, 0.081, 1.07	0.035, 0.092, 1.05
No. of reflections	1541	3452	2174	2490
No. of parameters	139	311	191	255
No. of restraints	8	21	17	8
Δρ_max_, Δρ_min_ (e Å^−3^)	0.50, −0.76	1.15, −0.98	0.27, −0.43	1.98, −1.49
Absolute structure	–	Flack *x* determined using 1096 quotients [(*I* ^+^) − (*I* ^−^)]/[(*I* ^+^) + (*I* ^−^)] (Parsons *et al.*, 2013 ▸)	–	–
Absolute structure parameter	–	−0.008 (6)	–	–

**Table:** 

	**21**	**22**
Crystal data		
Chemical formula	[Li_2_(C_6_H_4_O_3_)(H_2_O)_4_]	[Cs_3_(C_12_H_7_O_9_)(H_2_O)]·0.75H_2_O
*M*_r_	210.04	725.44
Crystal system, space group	Triclinic, *P* 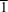	Triclinic, *P* 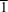
*a*, *b*, *c* (Å)	6.6971 (2), 8.1362 (3), 9.5658 (5)	7.7172 (3), 10.6962 (6), 11.3561 (6)
α, β, γ (°)	101.129 (4), 93.408 (3), 112.541 (4)	69.076 (5), 85.882 (4), 77.886 (4)
*V* (Å^3^)	467.21 (4)	856.07 (8)
*Z*	2	2
μ (mm^−1^)	1.15	6.41
Crystal size (mm)	0.19 × 0.10 × 0.02	0.24 × 0.09 × 0.06

Data collection		
Absorption correction	Gaussian (*CrysAlis PRO*; Rigaku OD, 2018–2021 ▸)	Analytical (*CrysAlis PRO*; Rigaku OD, 2018–2021 ▸)
*T*_min_, *T*_max_	0.661, 1.000	0.476, 0.711
No. of measured, independent and observed [*I* > 2σ(*I*)] reflections	5508, 1946, 1757	6129, 3567, 3349
*R*_int_	0.040	0.015
(sin θ/λ)_max_ (Å^−1^)	0.634	0.669

Refinement		
*R*[*F* ^2^ > 2σ(*F* ^2^)], *wR*(*F* ^2^), *S*	0.040, 0.110, 1.08	0.024, 0.051, 1.04
No. of reflections	1946	3567
No. of parameters	166	250
No. of restraints	9	11
Δρ_max_, Δρ_min_ (e Å^−3^)	0.33, −0.41	2.09, −1.49

Computer programs: *CrysAlis PRO* (Rigaku OD, 2018–2021 ▸), *SHELXT2018* (Sheldrick, 2015*a* ▸), *olex2.solve* (Bourhis *et al.*, 2015 ▸), *SHELXL2018* (Sheldrick, 2015*b* ▸), *OLEX2* (Dolomanov *et al.*, 2009 ▸), *CrystalMaker* (Palmer, 2020 ▸) and *PLATON* (Spek, 2020 ▸).
